# Risk management options to contrast the introduction of citrus fruit bacterial canker through ornamental Rutaceae in the Mediterranean Basin: An Italian case study

**DOI:** 10.1016/j.heliyon.2021.e06137

**Published:** 2021-02-06

**Authors:** Giuseppe Timpanaro, Arturo Urso, Alessandro Scuderi, Vera Teresa Foti

**Affiliations:** Dipartimento di Agricoltura, Alimentazione e Ambiente (Di3A), University of Catania, Italy

**Keywords:** Pest risk analysis, Stakeholders analysis, Import / Export of ornamental Rutaceae, Social, Economic and environmental criteria, Voluntary phytosanitary certification

## Abstract

Citrus bacterial canker (CBC) is a known disease caused by *Xanthomonas citri* subsp *citri*, which affects many species and varieties of Rutaceae. It causes evident damage on the epigeal parts of plant (leaves and branches) and, in particular, on the fruits, causing their fall and/or deterioration, making them unsuitable for sale. EPPO has signaled its presence in many Asian countries and in the Middle East, in South and Central America and in some regions of the African continent, but not yet in Europe. There are several possible ways of introducing this pathogen into the Mediterranean Basin and, among these, there is the trade of plant material for propagation and planting and the flow of tourism between the risk areas and the Mediterranean countries. This research demonstrates how the risk of invasion through ornamental Rutaceae is evident and identifies - in a participatory way through the involvement of stakeholders - some possible tools of phytosanitary protection. The methodological approach, with multi-criteria analysis, recognizes the interest in forms of protection represented by voluntary certification tools, rather than the introduction of new taxation that can finance the protection system.

## 1 Introduction

The spread of invasive species, as a consequence of the globalization and internationalization of markets, is a problem not only from a biological, ecological, environmental, and landscape point of view but also from an economic and social sides, widely analyzed in the literature [1, 2]. The “pest risk analysis” (PRA), which consists of “pest hazard identification” (PHI), “pest risk assessment” (PRA), “pest risk management” (PRM) and “pest risk communication” (PRC) has an interdisciplinary nature [3] because it must provide the scientific justification of the phytosanitary measures adopted by a country for its defense, in order to be able to demonstrate and compare the real necessity of the measures at world level [4]. Risk assessment and risk management interact, but they are also functionally separate risk analysis activities [5].

In the various areas of the PRA, according to a predominantly economic perspective [6], various contributions have been produced, not only on the assessment of the consequences deriving from the invasion of alien species in the form of direct damage produced or costs control, but also in understanding the problem from a holistic point of view; the provision of barriers and control policy tools (tariffs, import restrictions, etc.); the evaluation of the benefits and costs of control alternatives, also to increase the effectiveness and efficiency of publicly funded programs [7, 8]; the preparation of bioeconomic models that take into account the dynamics of interaction within and between ecological and economic systems [9, 10].

Since every invasion of alien species involves direct and indirect costs and, therefore, private and social costs [11], the economic impact assessments - necessary as scientific support provided by the International Plant Protection Convention and the World Trade Organization Agreement on the Application of Sanitary and Phytosanitary measures - are usually carried out with a qualitative or quantitative approach. The choice is often made to depend on the availability of data and the ability to prepare specific forecasting models, or able to justify the adoption of certain measures [12], or to support adequate decision-making strategies using the knowledge of the experts for the calibration of models and the evaluation of the potential distribution of an invasion [13]. However, the procedures used to assess impacts often influence the results of the assessment [14]; the “Cost-benefit analysis” is used for the purpose, among others, to evaluate the current and future costs and benefits in monetary units associated with a series of alternatives, projects or political instruments to combat environmental damage and the compromise of ecosystem services; the “Cost-Effectiveness Analysis”, for the comparison of different options or management options for the evaluation of the benefits of the control actions of invasive species; the “Multi-Criteria Analysis”, an approach that allows to incorporate multiple dimensions of the effects and to include both qualitative and quantitative information associated with the impacts of invasive species and those relating to the implementation of management responses, so as to overcome the uncertainties related invasion and decision making; the “Development Scenario”, in which the scenarios are plausible alternative descriptions, supported by quantitative indicators [15, 16].

Since the introduction paths of invasive species involve different goods, consumer goods, packaging, and multiple modes of transport, an international political framework was built, consisting of a series of commercial, environmental, and transportation agreements, subject to coordination and harmonization. Consequently, several international institutions interested in plant defense have arisen, such as the European Food Safety Agency (EFSA); the European and Mediterranean Plant Protection Organization (EPPO); the EU Directorate-General for Health and Food Safety (SANTE), the main body of the European Commission operating within the framework of the International Plant Protection Convention (IPPC) of the Food and Agricultural Organization (FAO) and the agreements signed in the framework of the World Trade Organization (WTO); the US Department of Agriculture Animal and Plant Health Inspection Service (APHIS). Institutional responses based on the models currently in use could include preventive measures to counter a possible invasion, management of the existing parasitic problem, or eradication. These actions consist in the regulation of production and trade to the point of involving the obligatory destruction of productive capital (for example, the abatement of production); loss of access to the export market; loss of recreational benefits for domestic consumers; losses for producers directly affected by the pathogen; even if in the long run it is possible to record the improvement of technology and company productivity and, in some cases, of product quality after reversing the effects of the pathogen [17].

In this context, it is necessary to consider Citrus Bacterial Canker (CBC), a potentially dangerous phytopathy for the Mediterranean Basin, caused by two similar but taxonomically distinct bacteria: *Xanthomonas citri* pv. *citri* (synonym *X. citri* subsp. *citri*) and *X. citri* pv. *aurantifolii* (synonym *X. fuscans* subsp. *aurantifolii*). As specified in other studies, “*X. citri*” is used to refer to any citrus fruit canker (hyperplasia) produced by *Xanthomonas*, whether Asian (*X. citri* pv. *citri*) or South American (*X. citri* pv. *aurantifolii*) groups [18, 19, 20, 21, 22].

Xc penetrates the plant tissue through stomas or wounds caused by cultivation techniques or natural agents such as wind or heavy rain. Within seven days of inoculation (although the incubation time is affected by temperature, increasing if below 20 °C), symptoms appear on the plant [23].

The CBC causes significant damage to the epigean organs of the plant (leaves and branches) and, in particular, on the fruits causing their fall and/or decay, making them not suitable for commercialization [23]. The disease has never been reported in Europe and the Mediterranean Basin and, therefore, the two responsible bacteria are included in the list of quarantine pathogens of the European and Mediterranean Plant Protection Organization (EPPO) [24]. However, the Panel on Plant Health (PLH) of the European Food Safety Authority (EFSA), in the document “Scientific Opinion on the risk to plant health of *Xcc* pv. *citri* and *Xcc* pv. *aurantifolii* for the EU territory” [25, 26], identified among the possible ways of entry of the bacterium into the Mediterranean Basin the trade flows of ornamental Rutaceae species, considering that some of these are widely cultivated in the Mediterranean countries and which activate a great economic and social function. The protective measures against the introduction of Xcc are governed by Directive 2000/29/EC and in the most recent EU Reg. 2031/2016, in which random CBC agents are inserted. In particular, the legislation acts to prevent many species of the genus “Citrus”, “Fortunella”, “Poncirus”, “Murraya könig” (actually since subject to *Diasphorina citri Kuway*) and related hybrids (subject to contamination), except for fruits and seeds.

The EU Implementing Regulation 2019/2072 provides a list of Rutaceae whose plants, subject to trade, can enter the EU territory with an "official declaration" of the Third Country or with the inclusion of a "supplementary declaration" on the phytosanitary certificate, certifying that the area of origin is free from Xc. Among the plants concerned are "Limonia L.", "Microcitrus Swingle", "Murraya J. Koenig", "Severina", "Poncirus Raf. and their hybrids", etc., although they are hardly traded from risk areas [22]. The risk, however, is connected to the import of plant material destined for planting, as *Xcc* would find favorable environmental conditions for its development, but not from the import of fruit due to the low probability of transferring the bacterium to a suitable host with this medium. Recent studies have shown that the risk of potential invasion is significant in light of the intense commercial exchanges of plant material between some areas at risk because the pathogen is present in them, and the Mediterranean Basin [22]; the consequent potential damage is, therefore, significant [27].

Since the presence of *Xcc* in the EU territory is not yet ascertained, it is, therefore, possible to definitively affirm that citrus cultivation enjoys a high degree of protection, by the institutional systems in charge in the various countries. These protection systems (phytosanitary authorities) act on the network intervening in the absence of phytosanitary documentation (passport for import/export activities) but do not prevent international trade since, in line with the WTO agreements, phytosanitary controls cannot become a real "non-tariff" barrier to free trade.

Rutaceous species used for ornamental purposes, for which the market is booming, are not covered. This leads to the conclusion that if a more restrictive system of protection against Citrus Bacterial Cancer was to be applied, it would have been necessary to include in the annexes of Directive 2000/29 EU and EU Reg. 2031/2016 the entire taxonomic category "Rutaceae family" and not the individual citrus species, because this choice meant more freedom for ornamental species.

Furthermore, since the literature shows that the invasion dynamics can be of the “intentional” and “unintentional” type, an adequate “pest risk assessment” and “pest risk management” in the case of the CBC must allow the identification of possible tools to prevent the risk of invasion within acceptable limits.

In this context, we asked the following research questions:

• How relevant is the Xcc problem for the Mediterranean Basin?
• Are the tools currently available adequate to protect EU borders?
• What protection tools can be adopted in addition to the existing ones?

This work has been developed to define, through the involvement of different categories of stakeholders and the use of multi-criteria analysis that allows considering complex problems, the aspects of the economic dimension of the problem of invasive species, the possible impacts of the CBC and defense strategies, to guide the activity of policymakers, public and private, in the choice of the most appropriate protection instruments. A possible invasion of CBC - through ornamental Rutaceae - generates in fact a diversified impact from an economic, social and environmental point of view, and since the means of combating it involve costs and benefits, they need a degree of acceptability in the different categories of stakeholders involved.

## 2 The role of multicriteria analysis and application fields in the “pest risk analysis”

**Figure 1:**
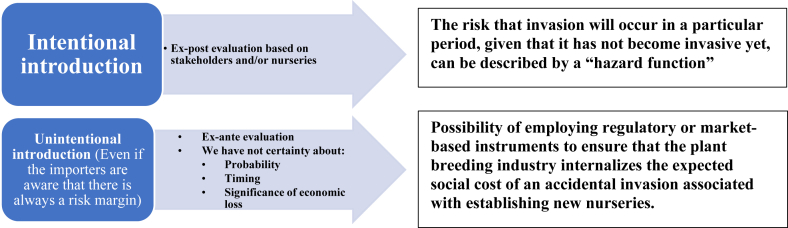
Spread of alien species (literature review, 2019).

Alien species can have several relevant ecological and socio-economic impacts and, therefore, effective and conflict-free management actions are needed, especially when the stakeholders who benefit from exotic species are different from those who bear the costs. Such conflicts of interest mean that management strategies are often not implemented unless all stakeholders of alien species or their management are involved [28]. In the invasion processes the potential role of commercial networks, air transport connections, geographical proximity, climate similarity, the biological wealth of the countries of origin and the tourist flow are highlighted [29]. The research carried out in the literature then allowed to distinguish different possible ways of diffusion of alien species (Figure 1):

The planning of any intervention on phytosanitary matters - for the great variety of the subjects involved - includes the management of a large quantity and quality of information and parameters with different levels of uncertainty and the need to build a dialogue between stakeholders, analysts, and scientists. For these purposes, the use of multicriteria decision analysis (MCDA), as a set of formal approaches, which seek to explicitly take into account multiple criteria in helping individuals or groups to explore the decisions that matter [30] is widespread [31].

The different methods (MAUT, AHP, ANP, PROMETHEE, ELECTRE, MAVT, NAIADE, TOPSIS, and DRSA, to name a few) are flexible, with various uncertainty management thresholds, a more or less demanding cognitive level for decision-makers, and they have more or less simplified software support in use [32, 33, 34, 35, 36].

In particular, models that implicitly use quantitative and qualitative information as a primary input for MCDA, to assist decision-making in the field of biosafety, have spread over the past two decades [35, 37, 38, 39, 40]. They are applied in the "pre-incursion quarantine" phase or in the "post-incursion control measures" phase. In the first case, the pre-raid quarantine is designed to reduce or eliminate the probability of a raid. It entails an increase in implementation and management costs, but also negative effects on the well-being of consumers caused by limited commercial flows, as shown by some partial equilibrium analyzes [41, 42]. In the second case, if the disease or a parasite/pathogen escapes the established quarantine measures (if any) and its entry is confirmed, there are three basic strategies for dealing with it, namely eradication, doing nothing, or a combination of these first options. The criteria used often provide for a reduction in the quantity of yield and an increase in the cost of production [43], also deriving from different decision-making protocols [44].

MCDA is so flexible that it is also used in cases where there are no specific risk maps (such as climatic suitability, host abundance or introduction potential) of invasive species [45], o defined invasive pathways, or an indication of sites more sensitive and susceptible to invasion [46]. Ecological-economic modeling offers a systematic and more objective way of organizing data and synthesizing knowledge [47].

Some studies incorporate stakeholders' views on invasion processes, combining expert analysis with information from fieldwork in an evaluation exercise. The management scenarios - assessed using the NAIADE model - are designed based on the available technical data, the perceptions of the stakeholders, the distribution of costs and benefits and the attribution of responsibility, in the event of an invasion of alien species capable to limit fishing in a body of water in the Caribbean Sea [48].

The process of involving stakeholders (actors) in the decision-making process, in management actions (co-design, co-creation, and co-implementation) and in the creation of knowledge on the effects of invasive alien species on biodiversity, on ecosystem services, on well-being human and local livelihoods is considered relevant to tackle the problems associated with biological invasions in a more holistic and successful way [49, 50, 51, 52]. The importance of the stakeholders is highlighted by the limited availability of resources for the management of invasive species, the impacts of the latter on the environment and economies, and the need to build univocal and traceable decisions [53].

MCDA has also been usefully used to define a compromise solution between sometimes divergent objectives. Indeed, it happens that decision-makers struggle to balance environmental objectives with other social objectives often in competition with each other, such as economic benefits and social well-being; on the other, uncertainty often prevails in understanding the invasion process and in communicating the risks of invasion to stakeholders [54].

The Deliberative Multi-Criteria Evaluation (DMCE) has been used to incorporate multiple social values and uncertainty into decision-making processes, combining the advantages of conventional multi-criteria decision analysis methods with the benefits of stakeholder participation to provide an analytical framework for evaluating complex multi-dimensional objectives. The DMCE process also ends up functioning as a risk communication platform where scientists, stakeholders, and decision-makers can interact and discuss the uncertainty associated with biological invasions [55].

## 3 Materials and methods

### 3.1 The MAMCA and the role of stakeholders in the MCDA

There are several multi-criteria models that successfully deal with the problem of managing environmental, economic, and social interventions or their political options since they offer solutions in this regard starting from different points of view and approaches. Each of them has advantages and disadvantages and, therefore, it is not possible to state that there is one method superior to the other. The choice of the multi-criteria approach to be applied is therefore subjective and, in particular, it is a choice that depends on the needs and requirements of the pest risk aspect to be analyzed. Hence, each method has certain properties; functions dependent and/or independent of the characteristics of the different criteria (qualitative and/or quantitative; with the same/different unit of measurement) taken into consideration; takes into account the subjective preferences of the stakeholders through utility functions or alternative systems; and may have been successfully applied to pest risk analysis problems in the past [56, 57, 58].

With these premises, an interesting approach for the “pest risk analysis” maybe that based on the multi-attribute method which is called Multi-Actor Multi-Criteria Analysis (MAMCA).

MAMCA is a method widely used in different application contexts to improve discussion among stakeholders, because it shows an evaluation of the different possible alternatives from the point of view of each interest, using the objectives of each as evaluation criteria. Therefore, both individual and group stakeholders are therefore not only those who influence a problem but also those who are affected, thus improving the procedural quality in the decision-making process [59, 60].

They are rooted in operational research and support for individual decisionmakers, but recently the emphasis has shifted towards multi-stakeholder processes to structure decision-making alternatives and their consequences, to facilitate dialogue on the relative merits of alternative action lines in the field of environmental and social impact assessment [61]. Indeed, the inclusion of stakeholder perspectives in environmental decision-making is a legal requirement in many countries and is widely considered advantageous as it can contribute to increasing the legitimacy of decisions, the probability of implementation and the quality of the results [62, 63].

The proposed use of MAMCA in the case of the CBC is ex-ante the invasion process, given that an integrated model for the evaluation of phytosanitary emergencies (against Citrus Tristeza Virus - CTV), economically, socially, biotic and phytosanitary sustainable the invasion process has already been tested ex-post by means of the Multi-Criteria Social Evaluation (SMCE). In this case, the "coexistence with the Citrus Tristeza virus" hypothesis is preferred, followed shortly after by the "total eradication and replanting" hypothesis, while the "abandonment or extirpation" hypothesis has assumed a marginal meaning [64].

### 3.2 Stakeholders involved and assessment questions in the Xcc pest risk analysis

Globalization and the growing integration of markets of goods, services, and production factors give rise to political, cultural, and environmental implications that are not without risks [65]. It has profoundly changed the movement of goods and people, both in terms of quantity and speed, has created new commercial routes, new modes of transport, new products and types of packaging and as a consequence, harmful organisms move by finding accommodation in goods, in means of transport, packaging, travelers' luggage, etc. [66, 67].

The risk of introduction of alien species varies according to the number of pathways (which depends on the number of countries in which the organism is present; on the number of plant hosts; on the number of marketing methods), on the volume of the product imported, the frequency of imports and the number of host species in the PRA area.

The parts potentially involved are, on the supply side of ornamental plants, different types of enterprises (nurseries, dealers, distributors, large-scale retail trade, etc.) and, on the demand side, the buyers - intermediate and final - of the product “ornamental rutaceae”. On the supply side - most of the subjects can be brought back within the category of “ornamental industry”, a multitude of productive firms with different products (not only ornamental rutaceae) and services offered according to the territorial areas of origin, the degree of company capitalization and the degree of integration with the upstream and downstream market (retail bedding and nursery stock; greenhouse/annuals; retail lawn and garden products; retail general merchandise; retail landscape materials; nursery container and field; landscape services/build; landscape architecture/design; wholesale bedding and nursery stock; retail garden equipment; wholesale landscape materials; retail florist and florist supplies; retail food and beverage; lawn and garden equipment; wholesale lawn and garden products; wholesale florist and florist supplies; wholesale garden equipment). To these must be added Academics, Collectors, General public, Landowners, Landscapers, Managers & policy makers, NGOs, Recreational users and State agencies.

Some structures operate on national and/or international markets from/vs EU third countries - intra EU (import/export), others only on local markets. The activities (production and/or trade and/or import of plants or plant products) internationally performed require - however - specific authorization issued by the official phytosanitary services and registration with the official Register of producers, so as to be able to release of the plant passport required by Directive 2000/29/Ce. The passport (in non-EU trade) and the phytosanitary certification (in intra EU trade), guarantee the health status of the goods handled, confirming their traceability, compliance with the appropriate conditions throughout the production and marketing chain and demonstrating the application measures to protect against the introduction into and the spread within the Community of organisms harmful to plants or plant products. Hence, the system is particularly protective, but some categories of subjects are exempted: among these, the occasional importers of small quantities of plants and their propagation material not destined for sale, and the “small producers”, (i.e. those who produce and sell vegetables and plant products their totality are intended as final use, within the local market, to people or buyers not professionally involved in the production of plants). These categories could represent a potential threat to the community.

Within this institutional scenario, an exploratory survey was conducted through a direct interview with professional nurserymen working in Sicily (Italy), at the center of the Mediterranean Basin [22], in order to acquire some elements of knowledge necessary for the design of a model of public intervention of prevention from the invasion of *Xcc* and some basic considerations have emerged:

• In this institutional system, governed by the legislation previously described, it is possible to affirm that it is unlikely that a living plant can escape checks, even if the prohibition does not exist for all ornamental rutaceae;
• the import/export of “live plants” from the areas of the planet in which it was currently reported the presence of *Xcc* appears limited to:
  ○ low market value of the generality of ornamental species and varieties (24–48 month plant) with wholesale prices ranging from € 6.50 for 17–18 cm pots, up to € 12.00–12.50 for 22–24 cm pots: very small figures to justify an intercontinental import, finding suitable cultivation conditions in the Mediterranean Basin;
  ○ difficulty in surviving plants for long-distance transport (2–3 weeks, on average), unless using high-cost unit means (air, controlled temperature and atmosphere, for example);
  ○ coexistence in the same areas of other types of plant diseases (for example HLB), which would imply for these products the accompaniment of the phytosanitary passport which would benefit, indirectly, also the protection from Xcc, analyzing Rutaceae plants to suitable diagnostic kits;
• there is currently a different degree of danger of invasion for:
  ○ type of nursery, with distinction between “professional” structure (both medium-large and micro-small size) recognized by phytosanitary services, registered in the regional register, authorized to issue extra EU passport and working with phytosanitary services for the issue of the intra-EU phytosanitary certificate. This structure is unlikely to be exposed to any importation at risk, in order to pursue additional profits. The professional operator, at certain levels of capitalization and productivity, can however delocalize its production activity in order to achieve greater economies of scale and scope, finding suitable environmental conditions and different phytosanitary situations in other areas of the planet. The situation is different for the amateur nursery, which experiences the derogation regime and which can be tempted by the possibility of widening its commercial offer, with imports at risk;
  ○ type of rutaceae species, with differences between common ornamental rutaceae (e.g. bitter orange, lemon, *Poncirus trifoliata*, *Citrus limonimedica*, kumquat or *Fortunella margarita*, *Citrus myrtifolia*, etc.), more easily found on the market of the Mediterranean areas, and more from niche (eg *Murraya paniculata*, *Citrus mitis*, *Coleonema pulchrum*, *Poncirus trifoliata*, *Zanthoxylum beecheyanum*, *Murraya exotica*), etc.;
  ○ type of trade, distinguishing between traditional and modern forms, represented by multi-channel, electronic and direct distribution formulas for final consumption. To date, the defense against invasion has been based on two conceptual elements, namely the traceability and control of the supply chain at a physical location. On the supply chain of ornamental plants nowadays and at different levels, electronic commerce is inserted in which the plant or part of the plant (scions, for example) skips some important elements of physical reference, in which the prescribed health checks can be carried out. The consumer can connect directly to platforms, not of production but of intermediation, which offer products of all kinds, even of clear foreign origin, community or non-EU, despite the EU Reg. 625/2017 on official controls. If these are not carried out cross-border, they should nevertheless be carried out starting from a site survey, which has not yet been completed;
• the risk degree of spread of *Xcc* is mainly linked to propagation material (slips, in particular), easily concealable (in the case of intentional invasion) and potentially able to escape customs controls (especially if placed inside apparent tourists). As it is known, it is possible to obtain seedlings from the multiplied marbles and, therefore, a multitude of plants that can meet one of the following destinies:
  ○ remain on the local market and potentially be able to spread *Xcc*;
  ○ export to the EU market, but in this case they will meet the phytosanitary certification (if to be involved is an authorized nursery, otherwise if the multiplication structure has an exemption, it is potentially able to spread *Xcc*);
  ○ export to extra-EU markets and, in this case, the plants go to the phytosanitary passport.

### 3.3 Model specification

Methodologically, a MAMCA has been done, which represents a wide family of techniques able to take into account at the same time a multiplicity of aspects typical of the problem that is being faced, both qualitative and quantitative, bringing out the different points of view of the actors involved.

Within the MAMCA, the evaluative function is expressed as follows:

V=f(O,C,A)

Therefore, the results of the evaluation (V), in the context of a determined decisional context, are a function of objectives (O), criteria (C) and alternatives (A).

**Figure 2:**
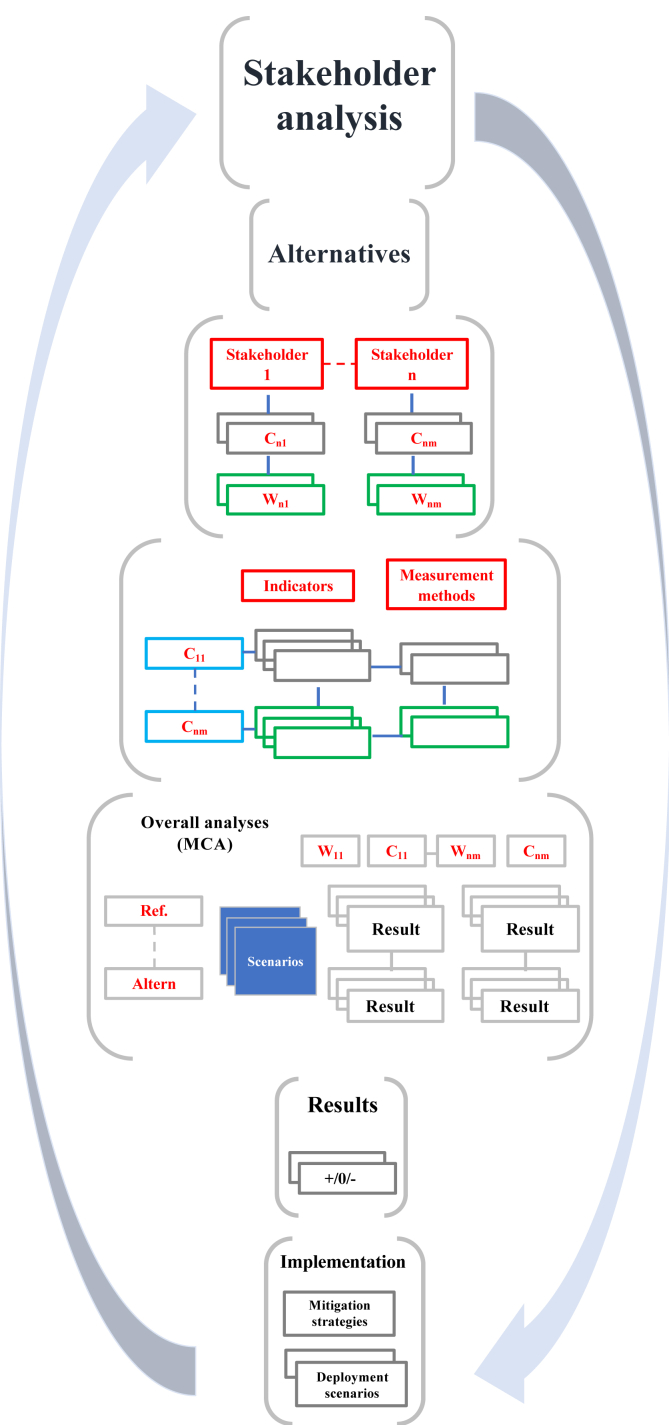
Structure of the MAMCA model for the assessment of the intervention to prevent the risk of invasion of Xcc (2019) (∗). (∗) Source: Our processing.

In detail, the proposed model (Figure 2), is based on:

• identification of the involved parties involved;
• definition of alternative scenarios;
• definition of the evaluation context, and decision criteria;
• assessment of the impact of alternative scenarios with respect to the criteria in question;
• final creation of the impact matrix.

The first step of the MAMCA approach consisted in defining the problem and identifying alternatives (phase 1). The explicit introduction of the stakeholders took place at a very early stage (phase 2); in fact, the stakeholders helped to identify the criteria, like the objectives and the weights, that is the importance attributed to the objectives previously identified (phase 3).

Stakeholders also had the opportunity to discuss alternatives or propose new ones (phase 1). In the fourth phase, for each criterion, one or more indicators were constructed (for example, direct quantitative indicators or scores on an ordinal indicator such as high/medium/low for criteria with values difficult to express in quantitative terms, and so on) (phase 4) and defined the measurement method, in order to measure the performance of each alternative in terms of contribution to the objectives of specific groups of stakeholders.

The overall preference scores for each option were determined with the weighted average of the scores for all criteria. Considering that the preference score for option *i* on criterion *j* is represented by *s*_*ij*_ and the weight for each criterion by ω_*j*_, for *n* criteria the overall score for each option is given by:

$$S_{i}=\;\omega_{1}s_{i1}+\;\omega_{2}s_{i2}+\cdots +\;\omega_{n}s_{in}=\;\overset{n}{\underset{j=1}{\sum }}\omega_{j}s_{ij}$$

The fifth step consisted in the construction of an evaluation matrix, which aggregated each alternative contribution to the objectives of all the stakeholders and then a classification of the various alternatives, which highlighted the strengths and weaknesses of the alternatives proposed (phase 6).

The stability of this ranking was assessed through a sensitivity and robustness analysis, to verify which variations of the model can generate substantial differences in the performance of the alternatives. To this end, the judgments of merit of some criteria have changed, to ascertain the degree of influence of each factor on the final decision.

The last phase of the methodology (phase 7) includes effective implementation.

### 3.4 Objectives, criteria, and alternatives adopted for the protection of the risk of invasion by Xc

The research was conducted in Sicily (Italy), selecting stakeholders among private subjects (professional operators and amateur operators, garden centers, landscape businesses and tertiary sector's companies, engaged in the production and marketing of ornamental Rutaceae) and institutional subjects (regional phytosanitary service, associations of citizens and scientific groups), in the composition shown by Table 1.

**Table 1:** Stakeholders sample composition (∗).

Stakeholders Category	Value %
**Private Subjects**	**82.2**
Professional Operators	26.7
Amateur Operators	8.9
Garden Centers	11.1
Landscape Businesses	13.3
Tertiary Sector's Companies, engaged in the production, plant propagation and marketing of ornamental Rutaceae	22.2
**Institutional Subjects**	**17.8**
Regional Phytosanitary Service	4.4
Scientific Groups	2.2
Associations of Citizens	11.1
**Overall**	**100.0**

(∗) Our elaboration.

A total of 45 stakeholders out of the 50 identified in the whole territory joined the survey. Their participation was requested for their specific expertise on the topic under investigation, for their work experience (as private or institutional subject) and for their knowledge of the legal and regulatory aspects of the "flowers and ornamental plants" sector. The final structure of the sample is sufficiently representative of the reality (for the Regional Phytosanitary Service were involved the managers of the 2 services active at regional level, in Palermo and Acireale (Sicily); for the Associations of Citizens were involved 5 regional associations representing hundreds of buyers of ornamental Rutacee; for the Professional Operators was found a sample equal to 5% of the active ornamental plants nurseries according to the register of operators authorized by the Regional Phytosanitary Service that alone intercept 30% of the related market of Rutacee; etc.), giving hope on the robustness of the analysis carried out.

The stakeholders were brought together through 2 workshops (organized in February 2019 and May 2019), each lasting 5 h, during which the aims of the research, the objectives, the importance of the role of the chosen stakeholders and the methods of participation were presented. All those present were given a specially prepared form-questionnaire and guaranteed anonymity and statistical confidentiality in accordance with the law in force.

During each workshop a discussion among the participants was activated, a discussion that was guided by the organizers to avoid the danger that some response was affected by the emergence of the behavior of one or more leaders, who impose their own personality. When this happened, the interviewer had the freedom to deepen, depending on the answers, the questions themselves and to return to certain aspects that in his opinion were important during the interview.

Therefore, the answers obtained are to be considered free from constraints and conditioning. The questionnaire contained free answer questions and closed questions, with Likert scale, for quantitative measurement.

The aim of the survey was to define a strategy for the defense against the invasion by XCC that could be replicated for the entire territory of the EU. Three hypotheses of intervention emerged from the discussion (Table 2).

**Table 2:** Hypotheses for the creation of a strategy to prevent the invasion of Xcc in the Mediterranean Basin (∗).

Scenarios	Detail of the proposal
Scenario/Hyp 1 “Status quo”	Maintenance of the “status quo”: the system self-manages, pending a complaint about the presence of Xcc in the Mediterranean Basin.
Scenario/Hyp 2 “Voluntary plant health certification”	Adoption of voluntary certification, by means of a protocol made public and approved by institutional bodies (research and phytosanitary certification) which includes a quarantine process.
Scenario/Hyp 3 “Taxation”	Forecasting of a tax - fixed or variable - for a commercialized product, in order to self-finance the system of prevention and sensitize consumers.

(∗) Our elaboration.

Ultimately, the proposal to extend the ban on imports to all rutaceae was renounced because it was seen as a heavy restriction on free trade, with penalties for the entire economy and society. Instead, two possible initiatives were held:

• the first (scenario/hypothesis 2) operates in the direction of achieving a greater degree of traceability of the ornamental product, in line with the recent EU Regulation 2031/2016 on measures to protect against pests harmful to plants (which in fact rewrites the Directive 29/2000) and Reg. 625/2017 on official controls, which will come into force on 14 December 2019;
• the second (scenario/hypothesis 3), on the other hand, constitutes a traditional measure for market regulation intervention (taxation), necessary to increase awareness of the problem and the resources necessary to face a possible emergent invasion with eradication programs, compensation, information to the consumer, training of operators. However, it is clear that the cross-border nature of the “*Xcc* invasion” problem implies that a coordinated approach is needed to tackle the issue beyond the political boundaries of a country.

To evaluate these three hypotheses, the evaluation criteria have been defined, representing the measurable aspect of the judgment that can characterize a dimension of the various choices taken into consideration. We preferred a more structured approach for determining weights and scores to be used in the multicriteria sorting analysis. This method can be used when the decision maker has difficulty in specifying the values relating to weights and scores (Table 3).

**Table 3:** Reference scale for comparisons in pairs in the strategy model of prevention from the invasion of *Xcc* in the Mediterranean Basin (∗).

Degree	Preference
1	Equally preferred
2	From equally to moderately preferred
3	Moderately preferred
4	From moderately preferred to highly preferred
5	Highly preferred
6	From highly preferred to very highly preferred
7	Very highly preferred
8	From very high preferred to extremely preferred
9	Extremely preferred

(∗) Our elaboration.

In particular, the ordering model requires that the involved stakeholders attribute a weight to each dimension to measure its relative importance and assign a score to different levers according to each dimension. Finally, levers are classified on the basis of their weighted average score.

In total, eighteen criteria or evaluation variables were used in the present case study (Table 4). These criteria were defined on the basis of the purpose and objectives of the evaluation of the analyzed case, which can be considered representative of the reality of Sicily but overall very similar to other areas of the Mediterranean Basin.

**Table 4:** Objectives and evaluation criteria adopted in the model of prevention strategy from the invasion of *Xcc* in the Mediterranean Basin (∗).

Goals	Weight of each criterion	Evaluation criteria	Weight of each sub-criterion
Economic	0.35	• product quantity	0.10
		• costs of prevention measures	0.10
		• costs of control measures	0.10
		• replanting costs and/or losses due to the need to grow replacement plants	0.15
		• production costs	0.25
		• producers earnings	0.20
		• effects on national and export markets and on prices paid	0.10
Environmental	0.30	• product quality	0.30
		• effects on parks and protected areas	0.20
		• effects on native plants, biodiversity and ecosystem services	0.25
		• effects on water quality, leisure time, tourism, landscape heritage	0.10
		• environmental restoration costs	0.15
Social	0.35	• increase in market prices	0.20
		• renounces the use of some species	0.30
		• lower recreational value	0.10
		• effects on employment	0.20
		• changes in domestic and foreign consumer demand	0.10
		• resources for research and advice	0.10

(∗) Our elaboration.

It is clear that goals and criteria refer to economic, social and environmental potential impact that the *Xcc* invasion could cause. In particular, the sub-criteria are linked to the aspect of company profitability (quantities produced, company revenues and profits, production costs and possible increases for prevention, monitoring, effects on target markets - with reference to the consequences of any impositions tax - any eradication costs, etc.); to the impact on the environment (potential consequences on biodiversity, modification of ecosystems, limitation to the use of protected areas, reduction of ecosystem services, etc.); the consequences on consumption and/or society (called to pay higher prices or to renounce the enjoyment of certain species and varieties, possible consequences on employment levels, etc.).

## 4 Results and discussion

### 4.1 CBC risk for the Mediterranean Basin

**Figure 3:**
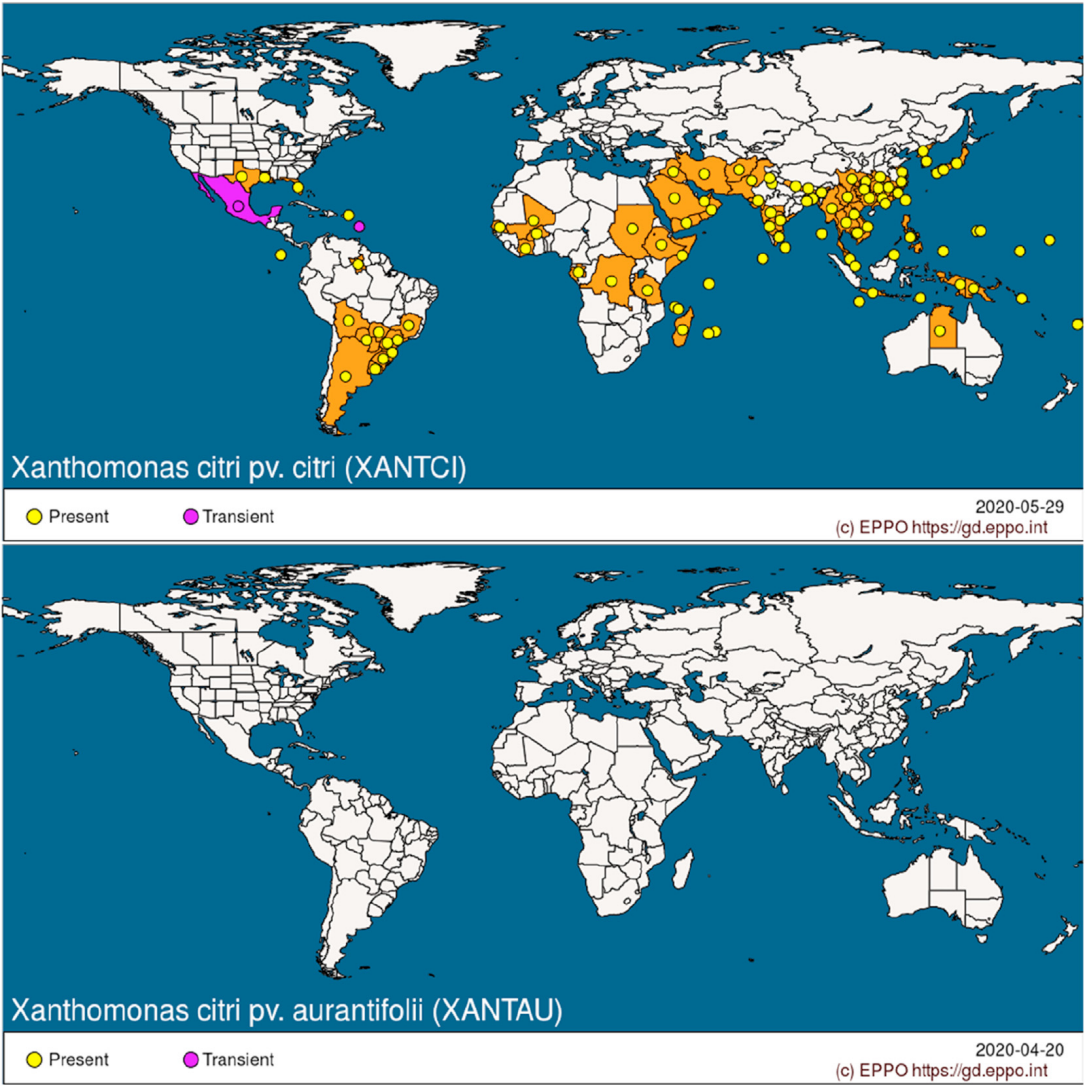
Diffusion of Xc sub. Citri and Xc sub. Aurantifolii in the world according to official statistics (∗). Source: EPPO database (accessed 2020).

According to the international plant health authorities (EPPO), Xc sub. Citri is present in various areas of the world and, in particular, in Africa (Somalia, Seychelles, Reunion, Mauritius, Mali, Ethiopia, Cote d’Ivoire, Congo, etc.), America (Bolivia, Brazil, Paraguay, Uruguay, Virgin Islands, Florida, Louisiana, etc.), Asia (Afghanistan, Bangladesh, China, India, Indonesia, Japan, Korea, Malaysia, Pakistan, Singapore, Sri Lanka, Vietnam, United Arab Emirates, etc.) and Oceania (Australia, Fiji, Micronesia, Papua New Guinea, Micronesia, etc.), as shown in Figure 3 (a). In the different areas, *Xcc* is classified by EFSA as “present” (“without detail”, “confirmed by investigation”, “widespread”, “under eradication”) or “transient”. In Europe, some surveys have shown the absence from Malta, Albania, Croatia, Cyprus, Netherlands and Turkey, so apparently the Mediterranean Basin is still exempt. As for *Xc* sub instead. *Aurantifolii*, this would not present, according to the EPPO, at the moment a phytosanitary problem (Figure 3, b), since the only suspect presence occurred in Netherlands, but a subsequent survey carried out in 2018 confirmed the absence of the bacterium.

The main climatic data relating to average temperatures and average rainfall in the two areas (countries in which Xcc was detected and the main countries of the Mediterranean Basin) shown in Table 5, although synthetic, demonstrate the existence of ideal conditions for the development of the phytopathy in certain contexts that thus become susceptible to hosting the spread of the pathogen, favoured by high temperature and humidity [22].

**Table 5:** Key climate-related information in areas where Xcc is present and in the main countries of the Mediterranean Basin (∗).

Country	Mean annual temperature (1901–2016)	Mean annual precipitation (1901–2016)	Country	Mean annual temperature (1901–2016)	Mean annual precipitation (1901–2016)
**Areas where Xcc ispresent**			**Main Mediterranean countries**		
Bangladesh	25.07 °C	2436.57mm	France	10.72 °C	831.61mm
Pakistan	20.01 °C	301.71mm	Spain	13.02 °C	607.50mm
China	6.39 °C	574.56mm	Turkey	11.17 °C	565.95mm
India	24.14 °C	1056.83mm	Greece	13.92 °C	679.08mm
Vietnam	24.22 °C	1793.17mm	Slovenia	8.72 °C	1375.73mm
Uruguay	17.50 °C	1203.84mm	Israel	19.48 °C	267.65mm
Thailand	26.33 °C	1553.58mm	Tunisia	19.42 °C	263.47mm
Argentina	14.31 °C	541.01mm	Albania	11.57 °C	1019.78mm
Malaysia	25.36 °C	3058.47mm	Algeria	22.71 °C	83.27mm
Indonesia	25.83 °C	2858.63mm	Cyprus	18.84 °C	483.89mm
Bolivia	20.93 °C	1093.41mm	Italy	11.86 °C	933.02mm
Brazil	24.96 °C	1741.79mm	Sicily	19.41 °C	673.80mm

(∗) Source: Climate Change Knowledge Portal. The World Bank. Accessed: december 2020. For Sicily, the data is taken from ISTAT, Rome.

The Mediterranean Basin is however home of intense commercial activity (import/export) in the sector of plants and parts of ornamental plants, as shown in some recent investigations [23, 26] which support the suspicion of the EFSA is the inclusion of *Xc* as quarantine organism in Europe, for which preventive measures must be taken to prevent the introduction of infected and asymptomatic plant material.

The size of this trade was determined through the UN ComTrade database, built by the United Nations Statistics Division (UNSD), which collects the commercial data of over 170 countries/territorial areas of the planet defined by FAO and the OECD. Inside, however, we do not find an item exclusively referring to ornamental Rutaceae, because they fall within the tariff code of the 8-digit combined nomenclature 06022090 called “Other” (NACE = *Nomenclature des Activités èconomiques dans les Communautés Européennes*). Not even EU Regulation 2018/1602, which modifies Annex I of Regulation (EEC) n. 2658/87 of the Council concerning the tariff and statistical nomenclature and the common customs tariff in force since 01/01/2019, solves this problem. Therefore, although the economic importance of the sector (equal to 21,858.54 mln €, EU - 28; 2018, source: EUROSTAT), the statistics on ornamental plants remain very aggregated, even if in 2012 Italy had requested at least the distinction between citrus fruit with edible and inedible fruit.

Therefore, the risk assessment of a potential invasion through the dynamics of international trade can, according to UN ComTrade, be carried out for code 06 (Live trees, plants, bulbs, roots, flowers, etc.). The item “cod. 06” although not exclusively referring to plants of the Rutaceae genus, since it includes all the plant material for non-food use, it is however significant for the definition of a commercial dimension at risk coming from countries/territorial areas in which *Xc* has been identified and present. In particular, the sub-codes are referred to:

• 0601: Bulbs, tubers, corms, etc., chicory plant (non-food);
• 0602: Live plants nes (*Not Elsewhere Specified*), roots, cuttings, mushroom spawn;
• 0603: Cut flowers, dried flowers for bouquets, etc.;
• 0604: Foliage etc except flowers for ornamental purposes.

**Figure 4:**
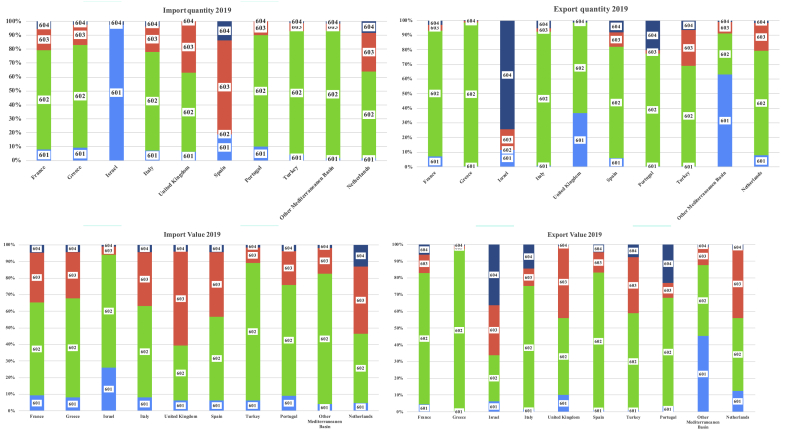
Structure of imports and exports of plant material by main EU and Mediterranean basin countries (2019) (∗). (∗) Source: Our processing on UNCOMTRADE data, subcodes 0601 = Bulbs, tubers, tuberous roots, corms, crowns and rhizomes; dormant, in growth or in flower; chicory plants and roots other than roots of heading. 0602 = Plants, live; n.e.s. in heading n. 0601 (including their roots) cutting and slips; mushroom spawn. 0603 = Flowers; cut flowers and flower buds of a kind suitable for bouquets or for ornamental purposes, fresh, dried, dyed, bleached, impregnated or otherwise prepared. 0604 = Foliage, branches and other parts of plants, without flowers buds, and glasses, mosses and lichens; suitable for bouquets or for ornamental purposes, fresh, dried, dyed, bleached, impregnated etc.

In the four subcodes as a whole, around 10,000 tons of plant material is imported into the Mediterranean Basin, for a total value of USD 47 million. European countries play a diversified role by type of product. Thus, France, Greece, Italy, Portugal, and Turkey are very active in the trade of live plants (up to 70% of total imports in Greece), unlike Israel and Spain much more exposed to commercial risk for bulbs, tubers, corms, etc (Figure 4). The different intrinsic value of the marketed plant material determines a certain variability in the import and export values. The intensification of trade inevitably triggers an increase in the demand for phytosanitary services and a tightening of the system of rules (sanitary, phytosanitary, and technical regulations) that governs commercial activities, with the risk of triggering international marketing bans and consequent compromises of benefits for consumers and profits for nurseries.

In fact, the same EFSA, in the scientific opinion of 2014, identifies:

• as many as seven possible ways of entering *Xc* through imports of plants or parts of plants for commercial or ornamental purposes, destined for their planting;
• some sources of risk linked to the careless behavior of tourists who are poorly informed about the health problems of plants and to the lack of awareness of amateur nurserymen;
• hopes for the “ornamental plants of Rutaceae”, the ban on the import of plants or parts of plants for their planting for commercial uses, even if not yet applicable to all Rutaceae;
• in quarantine facilities, before and after entry, a possible way to contain the spread of the infection, with variable intensity depending on the size of the shipments.

### 4.2 Pest risk analysis and definition of an appropriate level of phytosanitary protection

**Figure 5:**
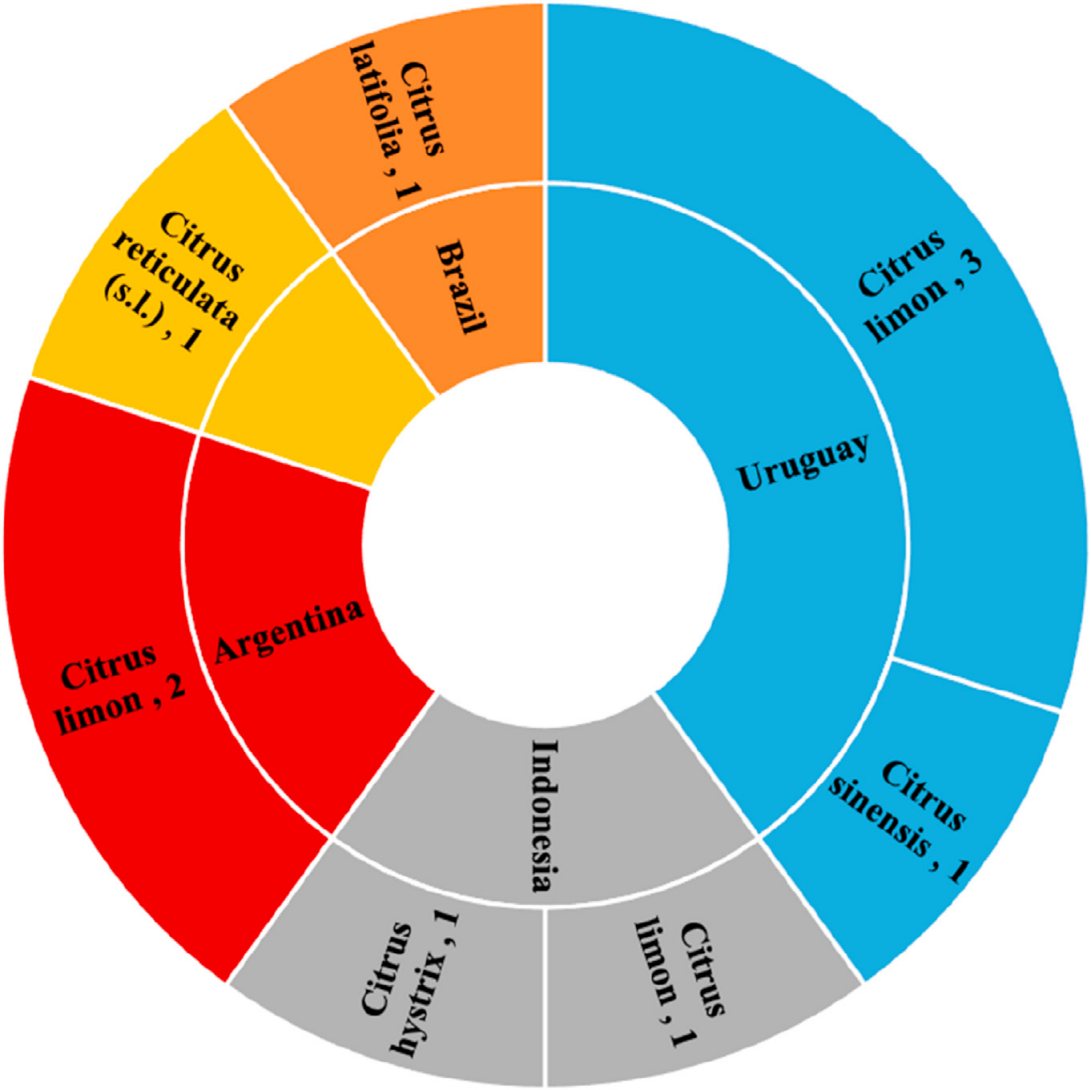
Interception of Xcc collected by European Union Notification System for plant Health Interception-EUROPHYT (2019).

A complete picture of the probability of spread of the pathogen causing bacterial citrus cancer, which is useful in defining possible protection options, can be obtained by considering the results of interceptions carried out by EUROPHYT (European Union Notification System for Plant Health Interceptions). This system promptly reports the interceptions detected by the phytosanitary systems spread in the European countries, in order to activate an alert to fight the spread of the different plant diseases. In 2019 alone, there are 10 active alerts on plant material (transplant or propagation) of different species of ornamental Rutaceae from the known areas with Xc spread (Figure 5).

Considering, on the one hand, the trade flow between areas at risk with Xc presence and areas in the Mediterranean Basin and, on the other hand, the interceptions made by EUROPHYT in European countries the probability of invasion is concrete and relevant, as shown in a recent analysis [22].

In this scenario, as an alternative to the “status quo”, the different possible intervention alternatives were evaluated to achieve a greater degree of protection from the invasion of *Xcc* (Table 6).

**Table 6:** Possible prevention interventions from the invasion of *Xcc* in the Mediterranean Basin and expected impacts (∗).

Type of impact	Status quo “0”	Ban to all Rutaceae	Mandatory plant passport	Mandatory plant health certification	Voluntary plant health certification	Quarantine
Impact on firms	None	Very high	High	None	High	High
Impact on consumers	None	Very high	High	Moderate	Moderate	Moderate
impact on institutions	None	Very high	none	High	high	High

(∗) Our elaboration.

Impact assessments take into account some possible negative effects on ornamental and commercial citrus producers (since a serious consequence of the invasion of *Xcc* is the transmission to commercial citrus fruits, with a considerable potential harm) of ornamental citrus consumers and commercial and related industries to support one and the other market.

For these reasons, the possibility of adopting a model, with some instruments of market control and not, to discourage a potential invasion of *Xcc* in the Mediterranean Basin has been evaluated. This is because, since there is currently no import ban for all ornamental *Rutaceae*, these represent for nurserymen an opportunity to differentiate the supply and increase their profits. Not only that, but the ban on the international commerce (import/export) of ornamental Rutaceae only for the risk that they can become an invasion vector involves high social costs, in the form of lack of benefits for consumers and profits for nursery owners. On the other hand, the intensification of trade in non-traditional species could increase the risk of the introduction and spread of Xcc in the Mediterranean Basin.

Prevention of the risk of introduction of *Xc* can be obtained through the qualification of nursery production according to standards that guarantee and protect the phytosanitary quality, as well as commercial. The certification of the origin of nursery materials represents a privileged way to guarantee efficiency and equity, fundamental functions of the wellness equation. In the countries of the Mediterranean Basin, however, there is also a diversity of situations in terms of partnerships with universities and phytosanitary centers, which can in a coordinated way promote the training of operators and the relative certification of skills, otherwise, the initiative is often limited exclusively to individual operators and their ethical behavior.

### 4.3 Results of the Xc risk prevention model

The model results provide important information on the perception of stakeholders of the problem of the invasion of *Xcc* and their preferences for a series of plausible political solutions (Table 7).

**Table 7:** Classification of scenarios corresponding to the highest consensus by type of stakeholders in the model of prevention strategy from the invasion of *Xcc* in the Mediterranean Basin (∗).

Stakeholders	Scenario “status quo”	Scenario “Voluntary plant heatlh certification”	Scenario “taxation”
Professional operators	0.86	0.79	0.18
Amateur operators	0.92	0.49	0.12
Garden centers	0.85	0.65	0.38
Landscape businesses	0.62	0.55	0.13
Phytosanitary services operators	0.36	0.39	0.28
Associations of citizens	0.29	0.85	0.91
Scientific groups	0.16	0.89	0.59
Tertiary sector's companies	0.54	0.84	0.21

(∗) Our elaboration.

Furthermore, the results indicate that perceptions and preferences regarding the development of policies to address the risks associated with the introduction of *Xcc* by means of ornamental Rutaceae vary between different groups of stakeholders, but also within the stakeholder groups.

In general, private operators (professionals, amateurs, garden centers, landscape businesses and tertiary sector companies) have shown a substantial convergence towards the rejection of new fixed or variable taxes, with limited assessment for the possible scenario. The ornamental Rutaceae are, in fact, an asset with elastic demand and a tax charge (both on the supply side and on the demand side) is seen as a reduction in price/quantity equilibrium, with the consequence that consumers pay more money and businesses receive less. In fact, various empirical evidence shows that both in case of sales tax and in tax on buyers there is damage for both, giving the parties identical regardless of who formally repays the tax. Not only that, but any product revenue is likely to be particularly low for the replacement of Rutaceae with other ornamental species, with fewer phytosanitary problems thus undermining the added value produced, the intense research activity upstream and the highly specialized factors (capital and work), who cannot find easily other jobs, especially in the short term. A basic concern emerges, therefore, that the taxation impairs the development of the market of ornamental Rutaceae.

For these operators, the “status quo” scenario is largely preferred, followed in order of importance by “voluntary certification”. In reality, the “status quo” scenario demonstrates the effectiveness of the current system of phytosanitary protection, not currently a presence of *Xcc* in the Mediterranean Basin, but this is not sufficient against a possible threat due to “moral hazard” behavior by non-professional subjects and/or “superficial” attitudes by passengers and tourists attracted by the “novelty” and by the desire to bring home an ornamental vegetable typical of Asian and Middle-Eastern areas. Therefore, the alternative to the expansion of the trade of plants and/or parts of plants and of the inflow of tourists remains in the perspective of offering ornamental productions with greater guarantee. On this last scenario appears a substantial convergence of opinions by professional operators, with some exceptions among amateurs.

The voluntary certification can be achieved by means of a voluntary protocol (codified process system verified and controlled by an independent third party) - which actually acts on the bureaucratic load of the production process, raising production costs - and/or creating a quarantine facility within the professional nurseries, so as to offer a commercial product (both for the domestic market and for the foreign market), which reassure on the many “qualities” of the company/process/product, including its traceability. A similar measure becomes effective even in the case where the producing company adopts decentralized production models, with relocations to other areas of the planet where the official requirements become weaker (i.e. areas where *Xcc* is present). Among other things, the voluntary supply of greater traceability should - as already mentioned - in line with the provisions of the new regulatory framework on phytosanitary surveillance (Regulation 2031/2016), starting from December 14 2019, which among other things provides for the possibility that professional operators will adopt a “Risk Management Plan related to harmful organisms”, plans that must be approved by the competent authority. The new legislation, however, appears to be strict with regard to the requirements for professional operators (not for example, on the total traceability - extended up to the cultivation substrates - and on the obligation to intervene), but with a view to supporting the free market exceptions to products (parts of plants not intended for sale) and non-professional operators, dangerous for a possible invasion of *Xcc*. These are “privileges” that amateurs do not intend to give up, as shown by the results of the model (these prefer to maintain the status quo).

**Figure 6:**
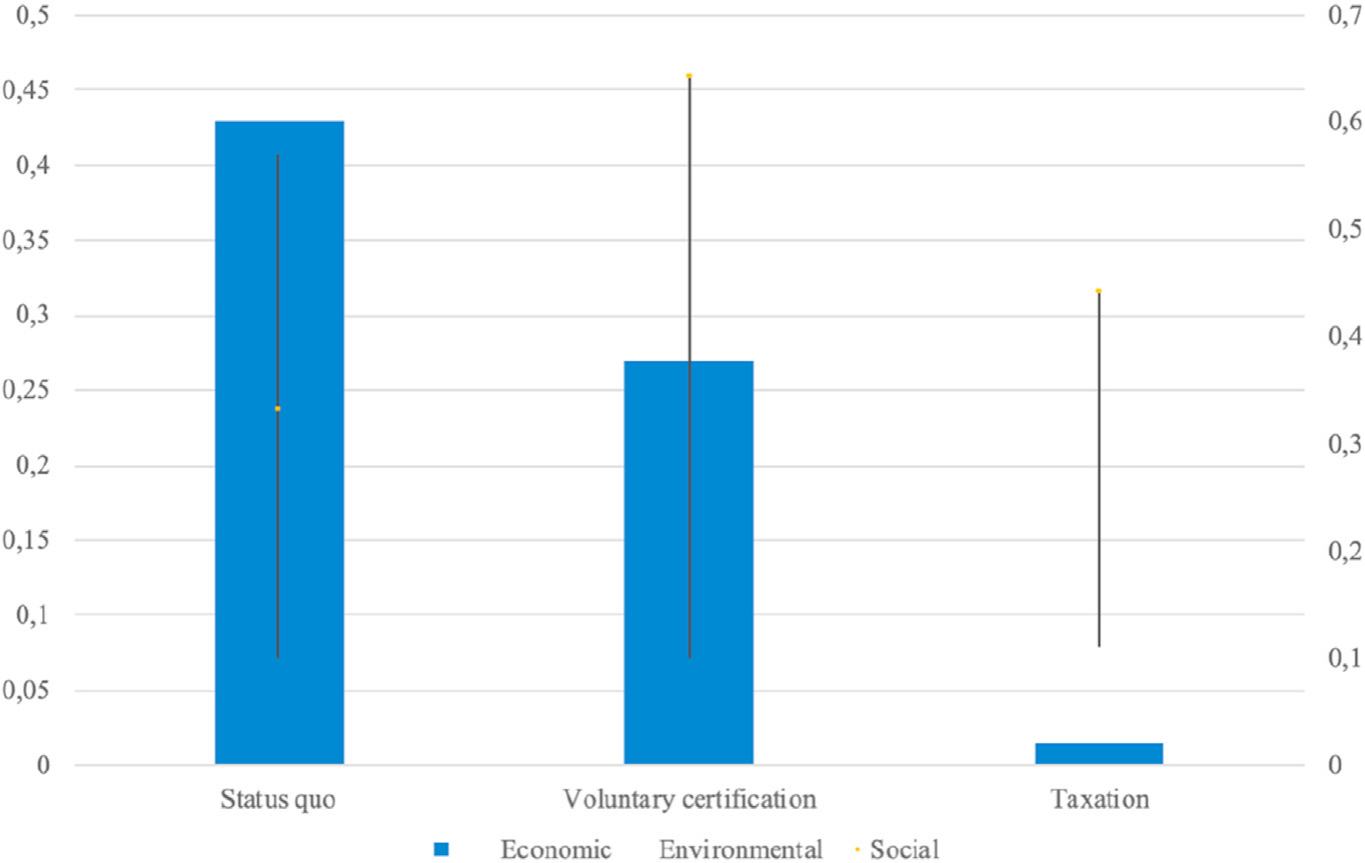
Summary matrix of the judgements expressed in the evaluation of Xc's protective measures (2019) (∗). (∗) Source: Our processing.

The sensitivity analysis shows the preference of the “status quo” by the production/consumption system, but also the transformation of opinions towards the affirmation of the “voluntary certification” due to the importance attributed to the environmental and economic criterion over the social one (Figure 6).

Finally, the model demonstrates the availability of consumers towards the payment of a “premium price” - also in form of taxation - that reassures the phytosanitary health of the purchased ornamental product. This choice is supported by a prior motivation towards a long-awaited and long-lasting recreational value and by an expectation of environmental protection and the landscape, which influenced the more or less recent information relaunched by the mass media on recent phytosanitary problems and damage produced in some territorial contexts (*Xylella fastidiosa*, *Citrus Tristeza Virus* or *CTV*, Red palm weevil from *Rhynchophorus ferrugineus*, etc.).

## 5 Conclusions

The identification of phytosanitary protection tools does not always represent an easy issue. It is necessary to find a balance system that does not compromise, on the one hand, the freedom of trade and all the advantages associated with it for the production and consumption system and, on the other, that safeguards the territories in their biological, environmental and naturalistic component and biodiversity protection [68].

The management of the protection of the Mediterranean Basin from *Xc*, where it can be conveyed by the areas in which the international bodies in charge (EPPO) have demonstrated its presence, through the commercial flow of ornamental Rutaceae and the flow of tourism, cannot be subtracted from these problems.

The recent regulatory revision and the advent of EU Reg. 2031/2016, has not only introduced improvements and different prescriptive effectiveness compared to the sector legislation in force in the past (European Directive 2002/89/EC), as well as higher levels of responsibility to professional operators but has retained some discretionary choices for member countries on the amateur nursery and the freedom of action of tourists.

Although not concerned with the latter, the introduction of potentially infected material by passengers depends not only from the intensity and clarity of the communication but also from the intensity of customs controls. The relative evaluation is difficult for the deficiencies in the phytosanitary surveillance staff with specific experience, for the deficiency in professional training and for cultural deficiency, since travelers are not fully aware of the socio-economic and environmental problem caused by a “superficial” import.

For this reason, some protection tools can be suggested in increasing information, in order to determine a cultural change, with the introduction of the obligation to declare “transport of plant material in baggage” to be presented to customs, with the definition of the “risk routes” and preparation of a dedicated control system. Attention should be paid to the implementing act of EU Reg. 2031/2016, which must clear the application content of art. 75 (on “exceptions for travelers' baggage”) which provides for the exemption of the phytosanitary certificate, but not of the inspection, for small quantities of certain species for non-professional use, excluding plant plants and certain plant products from any third country, an article that generates a certain ambiguity in its application in the various member countries.

As for the risk of import by the production/multiplication structures, the absence of *Xc* in Europe confirms the reliability of the institutional control system on *Rutaceae*. This is partly also the result of a favorable coincidence since *Xc* is present in 62% of the countries in which Huanglongbing is present (or HLB, *yellow branch disease* or *Citrus greening*) and, therefore, specific phytosanitary requirements are often associated with the use of the phytosanitary passport and quarantine procedures.

Ultimately, evaluation based on a multidimensional approach can contribute to the efficiency and effectiveness of the defense against the invasion of *Xcc* in the Mediterranean Basin, considered in its entirety: both in the preparation step, through the identification of resources, the objectives and possible choices, both in the decision-making phase, by choosing the most satisfactory solutions in the set of possible alternatives considered (operational implementation, flexibility and usability of the decisions to be taken).

The multi-criteria analysis carried out with the help of stakeholders has highlighted an interest in “voluntary certification” and the creation of a quarantine area within professional nurseries. This procedure, even if causing an increase in the bureaucracy of the production process and a consequent increase in production costs, could be a tool capable of reassuring the numerous “qualities” of the company/process/product, including product traceability and traceability.

The voluntary offer of greater traceability is in line with:

• the provisions of the new regulatory framework on plant health surveillance;
• with the strategic choices of the professional nursery company that in recent years has implemented high investments to diversify production, encouraging the affirmation of new production segments that have captured the interest of new consumers;
• with the need to protect the territory, ornamental Rutaceae and commercial species.

Voluntary certification also serves to overcome a shortcoming of the EU Implementing Regulation 2019/2072 that establishes uniform conditions for the implementation of EU Reg. 2016/2031, which in the case of Xc does not affect trade in "parts of plants intended for propagation (cuttings)".

Ultimately it is necessary to pursue:

• a greater collaboration between the Control Bodies;
• an improved operator training;
• the creation of a rapid alert system (TRACE - EUROPHTY, etc.);
• the definition of strict control procedures for electronic commerce and postal deliveries;
• the collection of research resources;
• the revision of the system of derogations for “small producers” (art. 65), also because of the EU regulation 2016/2031 leaves freedom to the Member States (for example, by committing them to guarantee the traceability of products/voluntary certification).

The limitations of the current research consist in the fact that the empirical verification was carried out exclusively in one area of the Mediterranean Basin, Sicily. Although this area has a central and strategic position in southern Europe for both the production and the marketing of the various species of ornamental rutaceae, it may not be exhaustive for the treatment of phytosanitary problems.

Future research developments will go in the both in the direction of verification of assumptions in other territorial contexts in the Mediterranean Basin (France, Spain and Turkey, at least) and in the direction of the setting up of a voluntary certification protocol, to create a strictly controlled supply chain starting from the propagation material, then to the mother plants up to the plants ready for sale. In this way it will be possible to promote the market of ornamental Rutaceae with the affirmation of diversified production and consumption models.

## Declarations

### Author contribution statement

Giuseppe Timpanaro; Vera Teresa Foti: Conceived and designed the experiments; Performed the experiments; Analyzed and interpreted the data; Wrote the paper.

Arturo Urso; Alessandro Scuderi: Performed the experiments; Wrote the paper.

### Funding statement

This work was supported by the ORPRAMed Project on “Risk assessment of introduction of *Xanthomonas citri* subsp. citri through commercial trade of ornamental rutaceous plants in the Mediterranean Basin”, ERA-NET ARIMNET 2-Call 2015 (dott.ssa Paola Caruso, Consiglio per la Ricerca in agricoltura e l'analisi dell’Economia Agraria–CREA, scientific coordinator). For the publication we thank 'Ateneo Founds 2020-2022, University of Catania, Open Access line'.

### Data availability statement

Data included in article/supplementary material/referenced in article.

### Declaration of interests statement

The authors declare no conflict of interest.

### Additional information

No additional information is available for this paper.
